# Determining minimum set of driver nodes in protein-protein interaction networks

**DOI:** 10.1186/s12859-015-0591-3

**Published:** 2015-05-07

**Authors:** Xiao-Fei Zhang, Le Ou-Yang, Yuan Zhu, Meng-Yun Wu, Dao-Qing Dai

**Affiliations:** 1 School of Mathematics and Statistics, Central China Normal University, Luoyu Road, Wuhan, 430079 China; 2 Intelligent Data Center and Department of Mathematics, Sun Yat-Sen University, Xingang West Road, Guangzhou, 510275 China; 3 School of Mathematics and Statistics, Guangdong University of Finance and Economics, ChiSha Road, Guangzhou, 510320 China; 4 School of Statistics and Management, Shanghai University of Finance and Economics, Guoding Road, Shanghai, 200433 China

**Keywords:** Protein-protein interaction network, Driver proteins, Controllability, Minimum dominating set, Centrality

## Abstract

**Background:**

Recently, several studies have drawn attention to the determination of a minimum set of driver proteins that are important for the control of the underlying protein-protein interaction (PPI) networks. In general, the minimum dominating set (MDS) model is widely adopted. However, because the MDS model does not generate a unique MDS configuration, multiple different MDSs would be generated when using different optimization algorithms. Therefore, among these MDSs, it is difficult to find out the one that represents the true driver set of proteins.

**Results:**

To address this problem, we develop a centrality-corrected minimum dominating set (CC-MDS) model which includes heterogeneity in degree and betweenness centralities of proteins. Both the MDS model and the CC-MDS model are applied on three human PPI networks. Unlike the MDS model, the CC-MDS model generates almost the same sets of driver proteins when we implement it using different optimization algorithms. The CC-MDS model targets more high-degree and high-betweenness proteins than the uncorrected counterpart. The more central position allows CC-MDS proteins to be more important in maintaining the overall network connectivity than MDS proteins. To indicate the functional significance, we find that CC-MDS proteins are involved in, on average, more protein complexes and GO annotations than MDS proteins. We also find that more essential genes, aging genes, disease-associated genes and virus-targeted genes appear in CC-MDS proteins than in MDS proteins. As for the involvement in regulatory functions, the sets of CC-MDS proteins show much stronger enrichment of transcription factors and protein kinases. The results about topological and functional significance demonstrate that the CC-MDS model can capture more driver proteins than the MDS model.

**Conclusions:**

Based on the results obtained, the CC-MDS model presents to be a powerful tool for the determination of driver proteins that can control the underlying PPI networks. The software described in this paper and the datasets used are available at https://github.com/Zhangxf-ccnu/CC-MDS.

**Electronic supplementary material:**

The online version of this article (doi:10.1186/s12859-015-0591-3) contains supplementary material, which is available to authorized users.

## Background

Proteins that are vital macromolecules rarely act alone. Diverse molecular processes within a cell are carried out by proteins through physically interacting with other partners. Therefore, protein-protein interactions (PPIs) are crucial for elucidating the structural and functional architecture of the cell [1,2]. Due to the development of high-throughput techniques, a large number of PPIs have been generated and accumulated, which paves the way for establishing the PPI networks [3,4]. To get a better understanding of the functional mechanism of PPI networks, determining driver proteins that are crucial for the control of the underlying network has become an important issue in systems biology [5].

The centrality-lethality rule suggests that the highly connected proteins in the PPI network are more likely to be essential [6]. The correlation between degree and essentiality was then conformed [7,8], and the reasons for this correlation were also examined [9-11]. Unlike degree centrality that counts the number of a node’s neighbors, betweenness centrality counts the number of shortest paths that pass through the node [12]. A node with high betweenness centrality has a large influence over the “information transfer” [13]. Therefore, high-betweenness proteins may act as important connectors in the network [14]. Previous studies suggest that the centrality index of a protein in a PPI network may be a good indicator of its biological importance and functional significance. However, as we know, there is still no systematic attempt to study whether the high-degree proteins or the high-betweenness proteins can offer full control of the underlying network.

**Figure 1:**
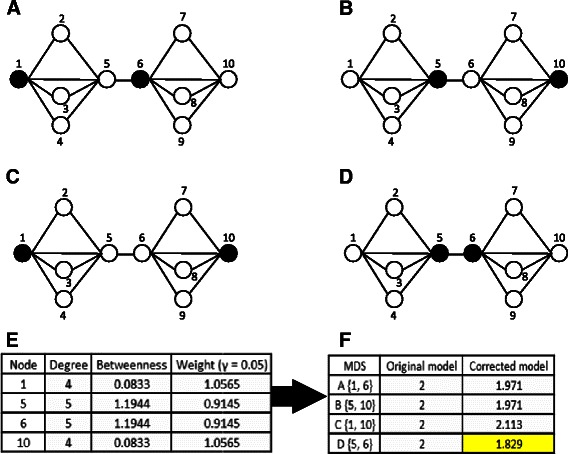
A graphical example that illustrates the motivations. This figure illustrates the concept of an MDS. Specially, an MDS is an optimized subset of proteins (black nodes) from which each remaining (i.e., non-MDS) protein (white nodes) can be immediately reached by one step. According to the standard MDS model, there exists four different MDSs for the given the toy network: **(A)** {1, 6}; **(B)** {5, 10}; **(C)** {1, 10}; **(D)** {5, 6}. Different optimization methods would generate different MDS configurations, and it is difficult to determine which one is reasonable in practice. For example, “lp_solve” produces result **(A)**; while “intlinprog” produces result **(B)**. To overcome this problem, we introduce a CC-MDS model of which the objective function takes variation in degree and betweenness centralities of proteins into consideration. In **(E)**, we compute the degree, betweenness and weight (with *γ*=0.05) defined in Equation (3) for the four proteins which seems to be driver proteins. In **(F)**, the objective functions of the original MDS model (Equation (1)) and the centrality-corrected model (Equation (2)) are computed. The objective function of the MDS model can not distinguish the four sets of proteins. According to the corrected model, **(D)** has the lowest value of objective function since the degree and betweenness of proteins 5 and 6 are highest. Both “lp_solve” and “intlinprog” determine proteins 5 and 6 as driver proteins.

In modern network science and engineering, the focus has been shifted to the identification of a minimum set of driver nodes that can control the entire network [15-20]. Recently, Liu et al. [15] made a ground-breaking contribution that predicted controller nodes using a maximum matching approach. Whereas their approach can only be implemented on directed networks. To apply on undirected networks, Nacher and Akutsu [21] addressed this problem from the perspective of minimum dominating set (MDS) [22]. In a network, a set of nodes is called a dominating set (DS) if all the remaining (i.e., non-DS) nodes can be reached by one link. The MDS is then defined as the smallest DS for a given network (see Figure 1). Inspired by the applications in telecommunications [23], Milenković et al. [24] developed two heuristic algorithms to detect dominating sets in PPI networks. They found that dominating sets are significantly enriched with biologically central genes. However, the dominating sets produced by their methods may be not minimal.

Recently, Wuchty [5] applied the MDS model developed by Nacher and Akutsu [21] on PPI networks and proposed some new metrics to evaluate the biological significance of the calculated minimum dominating sets. He found that the predicted driver proteins using the MDS model not only carry important functional characteristics (e.g., essential proteins, cancer-related proteins and virus-targeted proteins) but also play a key role to control the entire network (e.g., transcription factors and protein kinases). The promising biological significance of MDS proteins give new insight into the exploration of controllability in PPI networks. In the following text, we refer to the MDS model which is introduced in [21] and used in [5] as standard MDS model.

However, different optimization algorithms that are used to solve the MDS model may generate different configurations (see Figure 1A-D) [25]. Therefore, the MDS model does not create a unique driver set of proteins. To address this problem, Nacher and Akutsu [25] classified the nodes into three categories following the method of Jia et al. [26]: critical nodes that belong to every configuration, redundant nodes that never belong to any configuration and intermittent nodes that belong to some configurations but not all. In this procedure, we need to solve the MDS model *n* times, where *n* is the number of nodes. Therefore, compared with computing an MDS, their method needs much more CPU time. What is more, the biological significance of the three types of nodes is not analyzed in their study. Please note that the methods developed in [25] can be used to compute the critical nodes in directed networks too.

Recently, the MDS model have been widely extended and applied. For example, it was extended to address the controllability of bipartite networks [27]. Based on the framework of the MDS model, Nacher and Akutsu [28] introduced the concept of structurally robust control of complex networks where each node must be covered by at least two nodes in the dominating set. In this study, we consider a different extension of the standard MDS model to include heterogeneity in the degree and betweenness of proteins. Based on the assumption that high-degree and high-betweenness proteins are more likely to be controllers [5,21,25], we develop a degree and betweenness centrality-corrected MDS (CC-MDS) model. Despite its innocuous appearance, this corrected version turns out to have substantial effects.

We run both the standard MDS model and the CC-MDS model on three human PPI networks. Experiment results show that CC-MDS proteins (driver proteins determined using the CC-MDS model) predicted by different optimization methods are almost the same; while the overlap between MDS proteins (driver proteins determined using the MDS model) predicted by different optimization methods is quite low. We also observe that CC-MDS proteins are more important in maintaining the overall network connectivity than MDS proteins. Furthermore, compared with MDS proteins, CC-MDS proteins are more significantly enriched with high-degree proteins, high-betweenness proteins, multi-complex proteins, multi-functional proteins, essential genes, aging-genes, disease-associated genes, virus-targeted proteins, transcription factors and protein kinases. These results also indicate that the high-degree and high-betweenness proteins play an important role in controlling the underlying network. In the rest of this paper, we first review the standard MDS model. Then we introduce a degree and betweenness centrality-corrected version of the model, followed by a description of the biological data we use. The biological significance of MDS proteins and CC-MDS proteins are subsequently analyzed and compared from both topological and functional perspectives. Finally, we conclude the main contributions of this paper and give possible avenues for future works.

## Methods

### Minimum dominating set model

Given a PPI network, it could be represented by a graph *G*=(*V*,*E*), where *V* is the set of *n* nodes and *E* is the set of edges. An adjacency matrix *A* can be used to represent the edges, where *A*_*ii*_=1 (for convenience, a self-loop is also considered) for *i*=1,2,⋯,*n*, and *A*_*ij*_=1 if there exists an interaction between proteins *i* and *j* and *A*_*ij*_=0 otherwise.

A set *S*⊂*V* of proteins is considered to be a dominating set (DS) if every protein *v*∈*V* is either an element of *S* or adjacent to an element of *S* [5,21]. In other words, a DS is defined as a subset of proteins from which all the remaining (e.g., non-DS) proteins can be reached by one step. A minimum dominating set (MDS) is the smallest DS for a given network (see Figure 1A-D). For a given network, an MDS can be predicted as a minimum set of driver proteins [5]. To find out an MDS, each protein *i* is associated with a binary integer variable *x*_*i*_, where *x*_*i*_=1 represents protein *i* is an element of MDS and *x*_*i*_=0 otherwise. Mathematically, a DS needs to satisfy the following conditions $x_{i} + \sum _{j \in \Gamma (i)} x_{j} \ge 1$ for *i*=1,2,⋯,*n*, where *Γ*(*i*) is the set of neighbors of protein *i*. According to the definition of the adjacency matrix *A*, these conditions could be reformulated as: $ \sum _{j =1}^{n} A_{\textit {ij}} x_{j} \ge 1$, for *i*=1,2,⋯,*n*. Then the determination of an MDS that contains fewest members among all DSs can be formulated as the following binary integer-programming problem:

(1) $$ \left\{ \begin{array}{ll} \text{minimize} & \sum_{j=1}^{n} x_{j} \\ \text{subject \, to} & \sum_{j=1}^{n} A_{ij} x_{j} \ge 1 \, \, \, \, for \, i = 1, 2, \cdots, n. \\ & x_{j} \in \{0,1\} \, \, \, \, for \, j = 1, 2, \cdots, n. \\ \end{array} \right.   $$

This binary integer-programming problem can be solved using a branch-and-bound algorithm [29]. In this study, we implement the algorithm using two softwares: library “lp_solve” of the Matlab program language [30] and function “intlinprog” which is available in the Optimization ToolBox of MatLab version R2014b [31].

### Centrality-corrected minimum dominating set model

As mentioned in [25], there may exist more than one optimal solution to the binary optimization problem (1) for a given network. Therefore, MDSs identified by different optimization methods may be quite different (see Figure 1A-D). Because there are multiple MDS configurations, it is hard to determine which one is the real set that can control the entire network.

To overcome this problem, we take node degree and betweenness into consideration. This is because several recent studies have shown that network properties of biologically central genes exhibit some topological centrality compared to the rest of proteins in the PPI network and that centrality measures are discriminative in uncovering biologically central genes [5,24]. Based on the centrality-lethality rule [6], we assume that high-degree and high-betweenness proteins are more likely to be the controllers. Among all the MDS configurations, we would like to pick the MDS of which the members have the highest degree and betweenness (Figure 1D-F). However, it is difficult or impossible to compute all MDS configurations in practice. This is because we have no prior knowledge about the number of configurations and no effective methods to infer all configurations.

Instead of looking for all configurations, we consider a simple extension of the standard MDS model to include the heterogeneity in centrality. In the objective function of Equation (1), $\sum _{j=1}^{n} x_{j}$ is used to count the size of an MDS, where all nodes of the network are considered equally. To incorporate centrality heterogeneity, we introduce a centrality-corrected version $\sum _{j=1}^{n} \omega _{j} x_{j}$ to replace the original term, where *ω*_1_,*ω*_2_,⋯,*ω*_*n*_ are positive weights related to centralities of nodes. By doing so, we develop a centrality-corrected minimum dominating set (CC-MDS) model as follows:

(2) $$ \left\{ \begin{array}{ll} \text{minimize}& \sum_{j=1}^{n} \omega_{j} x_{j} \\ \text{subject \, to} & \sum_{j=1}^{n} A_{ij} x_{j} \ge 1 \, \, \, \, for \, i = 1, 2, \cdots, n.\\ & x_{j} \in \{0,1\} \ \ \ \ for \ j = 1, 2, \cdots, n. \\ \end{array} \right.   $$

Compared to the standard MDS model, the corrected model prefer to determine proteins with low weights as driver proteins.

Then we meet another question: what values of the weights will drive the model to identify high-degree and high-betweenness proteins? One possible way is that the weights are inversely proportional to the degrees and betweenness of proteins, i.e.,

(3) $$ \omega_{j} = \left({d_{j} b_{j}} \right)^{-\gamma},   $$

where *d*_*j*_ and *b*_*j*_ are the degree centrality and betweenness centrality of protein *j*, respectively; *γ*(≥0) is a parameter that controls the weights. When *γ*=0, the CC-MDS model (2) turns back to the standard uncorrected version (1); when *γ*>0, it prefers to pick high-degree and high-betweenness proteins (Figure 1E-F). This is because that high-degree and high-betweenness proteins will have lower weights than low-degree and low-betweenness proteins according to the definitions of weights, and therefore that picking high-degree and high-betweenness proteins will bring a smaller increment in the objective function of Equation (2) than picking low-degree and low-betweenness proteins. We will discuss the effect and choice of *γ* in the next section.

The CC-MDS model (2) is a weighted version of the MDS model (1); the weights changed the nature of the uncorrected model (1). This correction may seem minor, however, we will see in the next section that such correction has a big effect. Equation (2) is also a binary integer-programming problem, and can be also solved using library “lp_solv” and function “intlinpro”. Here we just replace a linear term by another linear term in the objective function. Therefore, the time complexity of computing a CC-MDS is the same as an MDS.

### Centrality calculation

Degree centrality is defined as the number of interacting partners of a protein. We compute degree centrality *d*_*j*_ of a protein *j* as $d_{j} = \sum _{i=1, i \ne j}^{n} A_{\textit {ij}}$.

Betweenness centrality is the number of shortest paths from all nodes to all others nodes that pass through the node. We determine betweenness centrality *b*_*j*_ of a protein *j* as $b_{j} = \sum _{i \ne j \ne k \in V} \frac {\sigma _{\textit {ik}}(j)} { \sigma _{\textit {ik}}}$, where *σ*_*ik*_ is the number of shortest paths between proteins *i* and *k* and *σ*_*ik*_(*j*) is the number of those paths that pass through protein *j*. We compute betweenness centrality using Matlab package “MatlabBGL” [32]. Furthermore, we normalize betweenness centrality by (*n*−1)(*n*−2)/2, where *n* is the total number of proteins in the network.

### Datasets

We use the high-quality protein interactions in H. sapiens from the High-quality INTeractomes (HINT) database that have considerable reliability and coverage (version: 06/03/2013) [33]. In terms of interaction type (binary or co-complex), we consider three separate data sets: binary interactions (binary), co-complex interactions (co-complex), and their combination (combined). For the sake of simplicity, we just use the largest connected component of each network. The properties of the PPI networks are listed in Table 1. We apply our model on the three PPI networks to determine driver proteins and use the following data sets to test the biological significance of the predicted driver sets of proteins.

**Table 1:** **Statistics of PPI networks and their corresponding sets of predicted driver proteins**

**Dataset**	**# proteins**	**# interactions**	**size**_**driver**_	***%***_**driver**_		
Combined	8,269	28,497	1,407	17.0		
Binary	7,865	24,368	1,393	17.7		
Co-complex	2,719	6,531	546	20.1		

We collect 1,846 manually determined protein complexes in H. sapiens from the Comprehensive Resource of Mammalian protein complexes (CORUM) database (version: February 2012) [34]. These complexes cover 2,556 proteins.

Gene Ontology (GO) annotations of H. sapiens proteins are downloaded from the GO database (version: 09/05/2014) [35]. GO annotations cover three domains: biological process (BP), cellular component (CC) and molecular function (MF). GO annotations with evidence codes IEA, ND and NAS are not considered. We also exclude annotations with NOT qualifier.

We use 2,501 essential genes in H. sapiens from the Database of Essential Genes (DEG) (version: 10.4) [36]. These data are collected from two literatures that identify human essential genes using comparative genomics analysis [37,38].

We collect 298 aging genes that are related to ageing from the Aging Gene (GenAge) Database (version: 03/05/2015) [39].

We collect disease-associated genes in H. sapiens from three public databases. We extract 3,003 proteins involved in human diseases from the Universal Protein Resource (UniProt) database using keyword “Disease [KW-9995]”, “reviewed:yes” and “organism: Homo sapiens (Human) [9606]”] (Version: 11/13/2014) [40]. We also collect the 3,094 disease-associated genes from the Online Mendelian Inheritance in Man (OMIM) database (version: 08/20/2014) [41]. In the “morbidmap” file, disorders with “[ ]”, “?”, “(1)”, “(2)”, “(4)” are excluded. Finally, we use 1,710 human genes relevant to disease phenotypes from the Genetic Association Database (GAD) (version: 04/19/2014) [42].

We obtain 704 human virus-targeted proteins from the Molecular INTeraction (MINT) database (version: 10/29/2012) [43]. Proteins that interact with human viruses are used as virus-targeted proteins.

We use 205 human transcription factors from the TRANSFAC database (version 7.4) [44] as provided by Molecular Signatures Database (mSigDB) [45]. We map the transcription factor matrix ids to gene symbols manually.

We obtain 514 protein kinases in human from the Regulatory Network in Protein Phosphorylation (RegPhos) database (version 2.0) [46].

For each dataset we use, we do the gene ID conversion (to gene symbols) according to HUGO Gene Nomenclature Committee (HGNC) [47]. We only consider proteins with known gene symbols in the experiments.

## Results and discussion

In the experiments, we apply both the MDS model and the CC-MDS model on three human PPI networks. For each model, we implement it using two optimization methods: “lp_solve” and “intlinprog”. Therefore, for each network, we can obtain four results: MDS-lp_solve, MDS-intlinprog, CC-MDS-lp_solve, CC-MDS-intlinprog. In the following text, we concentrate on analyzing the topological and functional significance of the predicted sets of driver proteins that correspond to the four results.

### Effect and determination of parameter

**Figure 2:**
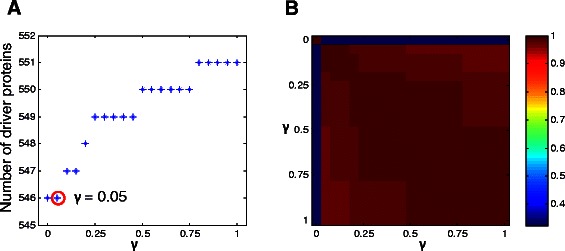
Effect of parameter *γ* on the resulting CC-MDS proteins for the intlinprog method in the combined network. In **(A)**, we present the effect of parameter *γ* on the number of predicted driver proteins. The x-axis denotes the value of *γ*; the y-axis denotes the number of driver proteins determined using the CC-MDS model; the red circle labels the value of *γ* we choose. In **(B)**, we present the overlap rate between the sets of driver proteins computed using different values of *γ*.

There is a parameter *γ* in the proposed CC-MDS model. To investigate the effect of *γ*, we wonder whether it has an influence on the number of determined driver proteins. To this end, we run the CC-MDS model on the three PPI networks with different values of *γ* (*γ*∈{0,0.05,0.1,⋯,1}). It clearly demonstrates that the number of determined driver proteins increases with the increasing of *γ* for the intlinprog optimization method (Figure 2A, Figures S1A and S2A in Additional file 1). According to the definitions of weights in Equation (3), picking higher-degree and higher-betweenness proteins will bring smaller increments in the objective function of our model (2). Therefore, before picking the lower-degree and lower-betweenness proteins, some higher-degree and higher-betweenness proteins may have been picked redundantly for large *γ*. This may be partly explain why more driver proteins are determined when *γ* increases.

We are then interested in the overlap of identified driver proteins for different values of *γ*. We find that the overlap rates (quantified using Jaccard coefficient) between the sets of CC-MDS proteins obtained with different values of *γ* (except for *γ*=0) are, on average, greater than 0.98 (Figure 2B, Figures S1B and S2B in Additional file 1). This indicates that the resulting sets of CC-MDS proteins are not very sensitive to the choice of *γ*. We also wonder whether the set of CC-MDS proteins obtained for a smaller value of *γ* is a full subset of the set of CC-MDS proteins obtained for a lager value. We experimentally find that even though the two sets overlap largely, the former set is not a full subset of the later set in some cases. Please note that the CC-MDS model equals to the standard MDS model when *γ*=0, therefore, the driver proteins predicted with *γ*=0 is very different from the ones predicted with other values of *γ*. The results for the lp_solve and intlinprog methods are comparable, therefore the data for the lp_solve method are not shown in the text.

One remain issue is to determine an optimal value of *γ* for a given biological network. Because the above results have shown that the choice of *γ* does not have a big effect on the resulting CC-MDS proteins, here we simply use a grid search method to determine its value. The chosen value needs to meet the following criteria: (1) it needs to be as large as possible such that the model tends to pick high-degree and high-betweenness proteins; (2) it needs to ensure that the size of resulting CC-MDS is equal to that of a standard MDS (e.g., *γ*=0). By doing so, we can ensure that the resulting CC-MDS is an optimal minimum dominating set of which the members have the highest degree and betweenness. Based on the two criteria, for both of optimization methods, we set *γ*=0.1 for the combined network, *γ*=0.15 for the binary network and *γ*=0.05 for the co-complex network (Figure 2A, Figures S1A and S2A in Additional file 1). In the following, unless otherwise stated, we mean the driver proteins detected by the CC-MDS model are obtained with these values.

### Overlap between driver proteins determined by different optimization methods

Before performing the overlap analysis, we count the number of determined driver proteins first. As aforementioned, a CC-MDS is also an MDS. Therefore, the MDS model and the CC-MDS model identify the same number of driver proteins with respect to both of softwares: “lp_solve” and “intlinprog”. That is the four models (MDS-lp_solve, MDS-intlinprog, CC-MDS-lp_solve, CC-MDS-intlinprog) produce the same of number of driver proteins for each network. Because the two optimization methods are not random, multiple runs of each model will produce a same result (This also apparently means that the number of identified driver proteins for each method for each network is constant). The results presented in Table 1 indicate that the corresponding predicted driver proteins involve fewer than 20% of all proteins. Therefore, a small set of proteins can control the entire network from the MDS perspective.

Both the MDS model and the CC-MDS model can be solved using algorithms “lp_solve” and “intlinprog”. Because they do not generate a unique driver sets, the two optimization methods may generate very different results. We wonder whether the CC-MDS model can improve the overlap rate between the sets of driver proteins computed using the two optimization methods. For this purpose, we use the Jaccard index to quantify the overlap rate. A larger value of overlap rate indicates that more common driver proteins are determined by the two optimization methods. We calculate overlap rates for both the MDS mode and the CC-MDS model. We observe that the overlap rates of CC-MDS proteins are higher than those of MDS proteins on all the three networks (Table 2). The overlap rates between MDS proteins determined using “lp_solve” and “intlinprog” are around 0.5. On the contrary, the corresponding overlap rates of CC-MDS proteins are close to 1, which shows that the CC-MDS proteins computed using different methods are nearly the same. Therefore, the overlap rate between the sets of drivers proteins computed using different optimization algorithms can be increased considerably by taking heterogeneity in centralities of proteins into consideration.

**Table 2:** **Overlap rate and the number of common members between the sets of predicted driver proteins computed using “lp_solve” and “intlinprog”**

**Dataset**	**MDS**	**CC-MDS**
Combined	0.5219 (965)	0.9958 (1,404)
Binary	0.4397 (851)	0.9971 (1,391)
Co-complex	0.4388 (333)	1.0000 (546)

### Degree distributions of determined driver proteins

**Figure 3:**
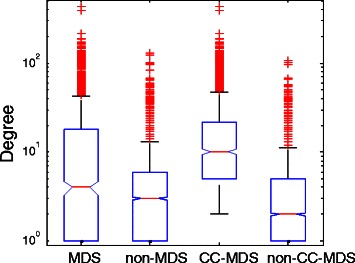
Degree distributions of the predicted driver and non-driver proteins for the intlinprog method in the combined network. The degree distributions are represented by box plots (line = median).

The centrality-lethality rule reveals that high-degree proteins tend to be more essential than low-degree proteins [6]. We wonder whether MDS proteins and CC-MDS proteins are enriched with high-degree proteins. From Figure 3 and Figure S3 in Additional file 1, we observe that the degrees of driver proteins (determined using both MDS and CC-MDS) are, on average, larger than those of non-driver proteins. Furthermore, the CC-MDS model targets more high-degree proteins than the MDS model. The Wilcoxon test is implemented to test the significance of the difference between degree populations of predicted driver proteins and non-driver proteins. The results presented in Table S1 in Additional file 1 show the statistical significance. In addition, the lower p-values of CC-MDS proteins show much stronger significance. Therefore, compared with MDS proteins, CC-MDS proteins are more central in the networks.

### Betweenness distributions of determined driver proteins

**Figure 4:**
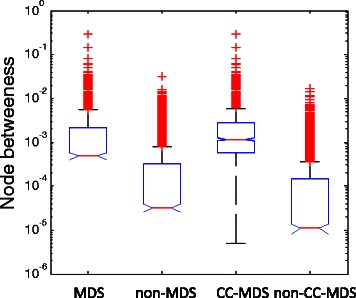
Betweenness distributions of the predicted driver and non-driver proteins for the intlinprog method in the combined network. The betweenness distributions are represented by box plots (line = median).

Node betweenness centrality is an indicator of a node’s central role in a network [12]. Proteins with high betweenness centralities have a large influence on the information transfer in the PPI networks [14], suggesting that the betweenness of driver proteins may be higher than those of non-driver proteins. We find this assumption is well verified by both MDS proteins and CC-MDS proteins (Figure 4 and Figure S4 in Additional file 1). We also observe that CC-MDS proteins are enriched with more high betweenness proteins than MDS proteins, which is reflected by the lower p-values (Wilcoxon test) of CC-MDS proteins (Table S2 in Additional file 1). These results indicate that CC-MDS proteins are more likely to be important connectors that link the entire network than MDS proteins.

### Vulnerability to attack against determined driver proteins

**Figure 5:**
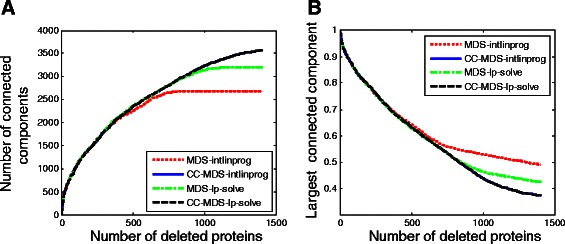
Vulnerability to attack against the predicted driver proteins for the combined network. Starting with the most connected proteins, the proteins are successively deleted, and **(A)** the number of connected components and **(B)** the size of largest connected component after each deletion is calculated. There is one curve for each set of predicted driver proteins that shows **(A)** the number of connected components and **(B)** the fraction of nodes in the largest connected component as a function of the number of deleted proteins.

Proteins that play a role in maintaining the overall connectivity of network may have a big impact on network’s resilience. We wonder whether CC-MDS proteins are more important in maintaining the overall connectivity than MDS proteins. Therefore, we measure this impact by performing a robustness analysis in a similar manner to [5] and [10]. For each set of driver proteins determined using different models, we sort the members according to their degree. Starting from the highest degree proteins we successively delete proteins and calculate the number of connected components and the size of the largest connected component in the altered network. A protein set determined by a method that generates more connected components or produces a smaller largest connected component is more disruptive. For all the three networks, CC-MDS proteins have a higher impact on the resilience of the underlying network in terms of both the number of connected components (Figure 5A and Figure S5 in Additional file 1) and the size of the largest connected component (Figure 5B and Figure S6 in Additional file 1).

### Enrichment analysis of multi-complex proteins

**Figure 6:**
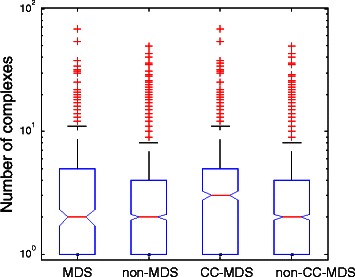
Distributions of the number of associated complexes of predicted driver and non-driver proteins for the intlinprog method in the combined network. The distributions are represented by box plots (line = median).

Proteins are often involved in more than one complex to serve different biological functions over different stages of cell cycle [48] or in different tissues [49]. Proteins shared by multiple complexes may play essential roles in multiple cellular processes. To investigate the importance of the determined driver proteins, we expect that MDS proteins and CC-MDS proteins may appear in more complexes than non-MDS proteins and non-CC-MDS proteins. We use the protein complex data from the Comprehensive Resource of Mammalian protein complexes (CORUM) database [34]. Figure 6 and Figure S7 in Additional file 1 shows that the predicted driver proteins appear in more protein complexes than non-driver proteins and that CC-MDS proteins clearly belong to more complexes than MDS proteins. The results are statistically significant according to Wilcoxon test (Additional file 1: Table S3). Therefore, CC-MDS proteins are more central in the network to reach other proteins in different complexes than MDS proteins.

### Enrichment analysis of multi-functional proteins

**Figure 7:**
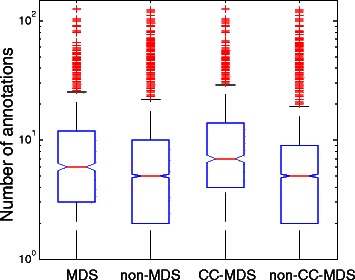
Distributions of the number of associated BP annotations of predicted driver and non-driver proteins in the combined network. The distributions are represented by box plots (line = median).

Multi-functional proteins have multiple domains to interact with distinct sets of partners, each serving a different molecular function [50,51]. Proteins that perform multiple roles are important for cell’s functional organization [52]. We wonder whether predicted driver proteins involve more functions than non-driver proteins. We perform the experiment using Gene Ontology annotations from the Gene Ontology database [35]. From Figure 7 and Figures S8-S10 in Additional file 1, we observe that the predicted driver proteins are associated with more GO annotations than the non-driver proteins for all the three subontologies (BP, CC, and MF). In addition, CC-MDS proteins participate in, on average, more functions than MDS proteins. Table S4 in Additional file 1 shows the statistical significance of the difference between populations of the number of annotated GO annotations of driver proteins and non-driver proteins (Wilcoxon test). The lower p-values of CC-MDS proteins show that the CC-MDS model can detect more multi-functional proteins than its uncorrected version.

### Enrichment analysis of essential genes

Essential genes are genes that are critical for the survival of the organisms [53], suggesting that the predicted driver proteins may be enriched with essential genes. Using the essential genes obtained from the Database of Essential Genes (DEG) [36], we perform an enrichment analysis by applying Fisher’s exact test. We observe that essential genes are significantly enriched in both MDS proteins and CC-MDS proteins for all the three networks (Table 3). More importantly, we find that CC-MDS proteins can recover more essential genes than MDS proteins and that the sets of CC-MDS proteins show much stronger enrichments of essential genes than the sets of MDS proteins.

**Table 3:** **Enrichment of the predicted driver proteins among essential genes**

	**intlinprog**		**lp_solve**	
**Dataset (# proteins)**	**MDS**	**CC-MDS**	**MDS**	**CC-MDS**
Combined (1,712)	2.3E-04 (343)	1.4E-22 (432)	2.0E-17 (413)	1.4E-22 (432)
Binary (1,632)	5.1E-06 (353)	1.8E-20 (421)	6.1E-16 (404)	1.8E-20 (421)
Co-complex (920)	8.5E-03 (211)	4.3E-15 (264)	7.1E-09 (243)	4.3E-15 (264)

### Enrichment analysis of aging genes

Aging genes are human genes implicated in the process of aging, therefore they are one type of human biologically central genes. To reveal the biological significance of the resulting minimum dominating sets, we wonder whether these sets are significantly enriched with proteins that govern longevity. After collecting 298 aging genes from the Aging Gene (GenAge) Database [39], we apply Fisher’s exact test to evaluate the statistical significance. We find that ageing-related genes are indeed significantly enriched in the sets of CC-MDS proteins and the sets of MDS proteins (Table 4). In addition, the CC-MDS model can capture more aging genes than the standard MDS model, which shows the effect of degree and betweenness centralities on the problem of diver protein detection.

**Table 4:** **Enrichment of the predicted driver proteins among aging genes**

	**intlinprog**		**lp_solve**	
**Dataset**	**MDS**	**CC-MDS**	**MDS**	**CC-MDS**
**(# proteins)**				
Combined (259)	1.2E-10 (86)	5.8E-21 (107)	5.0E-16 (98)	5.8E-21 (107)
Binary (254)	1.5E-07 (79)	1.1E-18 (104)	1.3E-13 (94)	1.1E-18 (104)
Co-complex (206)	1.8E-07 (72)	3.2E-19 (96)	3.4E-12 (83)	3.2E-19 (96)

### Enrichment analysis of disease genes

A genetic disorder is caused by one or more abnormalities in the genome. Genes associated with diseases have special biological roles in the cell [54]. Assuming that driver proteins may significantly contribute to human genetic disorders, we expect that proteins that govern diseases may significantly appear in MDS proteins and CC-MDS proteins. We collect disease-associated genes from three public databases: Genetic Association Database (GAD) [42], Online Mendelian Inheritance in Man (OMIM) database [41] and Universal Protein Resource (UniProt) database [40]. From Table 5, we find that disease-related proteins are significantly enriched in both the sets of MDS proteins and CC-MDS proteins on all the three networks (Fisher’s exact test). Furthermore, the sets of CC-MDS proteins include more disease-related genes than those of MDS proteins, of which the statistical significance can be validated by the lower p-values of CC-MDS proteins.

**Table 5:** **Enrichment of the predicted driver proteins among disease-associated genes**

		**intlinprog**		**lp_solve**	
**Database**	**Dataset (# proteins)**	**MDS**	**CC-MDS**	**MDS**	**CC-MDS**
	Combined (1071)	6.7E-04 (222)	2.3E-14 (274)	2.2E-08 (249)	2.3E-14 (274)
GAD	Binary (1020)	1.1E-02 (210)	5.6E-14 (270)	1.5E-09 (252)	5.6E-14 (270)
	Co-complex (487)	1.5E-02 (118)	3.2E-08 (144)	3.6E-05 (132)	3.2E-08 (144)
	Combined (1639)	4.7E-07 (349)	2.0E-12 (378)	5.2E-08 (355)	1.1E-12 (379)
OMIM	binary (1558)	2.0E-02 (308)	5.5E-10 (362)	5.9E-08 (351)	3.4E-10 (363)
	Co-complex (657)	3.0E-03 (159)	3.2E-07 (179)	2.0E-05 (171)	3.2E-07 (179)
	Combined (1686)	2.5E-05 (346)	2.9E-09 (371)	1.9E-06 (354)	1.9E-09 (372)
Uniport	binary (1604)	1.6E-03 (328)	5.5E-08 (360)	5.9E-07 (354)	3.6E-08 (361)
	Co-complex (711)	6.5E-03 (168)	4.3E-05 (181)	8.8E-04 (174)	4.3E-05 (181)

### Enrichment analysis of virus-targeted proteins

Besides genetic diseases, there also exists virally implicated diseases that are associated with viral infections [55]. Human viruses seize certain proteins to control a host cell [56], suggesting that virus-targeted proteins may be significantly enriched in the sets of predicted driver proteins. We use the 704 proteins that interact with at least one human viruse from the Molecular INTeraction (MINT) database [43]. Applying Fisher’s exact test, we find that virus-targeted proteins significantly appear in both MDS proteins and CC-MDS proteins (Table 6). We also observe that the CC-MDS model can identify more virus-targeted proteins than the MDS model.

**Table 6:** **Enrichment of the predicted driver proteins among virus targeted proteins**

	**intlinprog**		**lp_solve**	
**Dataset (# proteins)**	**MDS**	**CC-MDS**	**MDS**	**CC-MDS**
Combined (591)	3.8E-06 (143)	2.2E-17 (181)	7.9E-09 (154)	2.2E-17 (181)
Binary (575)	6.4E-04 (133)	8.5E-14 (172)	1.4E-09 (158)	8.5E-14 (172)
Co-complex (344)	5.2E-02 (83)	3.8E-10 (115)	1.1E-07 (108)	3.8E-10 (115)

### Enrichment analysis of transcription factors

Transcription factors are important proteins that control the rate of transcription of genetic information from DNA to messenger RNA. Therefore, transcription factors play crucial roles in regulation of gene expression [57]. To show the biological significance of the predicted driver proteins, we need to make sure whether such sets are significantly enriched with transcription factors. After collecting 205 human transcription factors from the TRANSFAC database [44], we apply Fisher’s exact test to assess the statistical significance. We experimentally find that transcription factors significantly appear in MDS proteins and CC-MDS proteins (Table 7). However, the sets of CC-MDS proteins cover more transcription factors than the sets of MDS proteins, except in the binary network.

**Table 7:** **Enrichment of the predicted driver proteins among transcription factors**

	**intlinprog**		**lp_solve**	
**Dataset (# proteins)**	**MDS**	**CC-MDS**	**MDS**	**CC-MDS**
Combined (155)	1.7E-02 (38)	2.5E-06 (50)	2.0E-04 (45)	2.5E-06 (50)
Binary (148)	4.5E-03 (40)	2.9E-04 (44)	2.9E-04 (44)	2.9E-04 (44)
Co-complex (99)	2.9E-02 (29)	1.6E-04 (36)	2.9E-02 (29)	1.6E-04 (36)

### Enrichment analysis of protein kinases

Protein kinases that catalyze protein phosphorylation play crucial regulatory roles in intracellular signal transduction [58]. Therefore, we investigate whether protein kinases significantly appear in the sets of predicted driver proteins using Fisher’s exact test. We obtain protein kinases from the Regulatory Network in Protein Phosphorylation (RegPhos) database [46]. It can be clearly seen from Table 8 that protein kinases are significantly enriched in CC-MDS proteins. For the MDS model, the deriver proteins computed using library ‘‘intlinprog’’ from the combined and co-complex networks are not statistical significance (p-value > 0.01). After incorporating heterogeneity in the centralities of proteins into the standard MDS model, the CC-MDS model can recover more protein kinases than the uncorrected version.

**Table 8:** **Enrichment of the predicted driver proteins among protein kinases**

	**intlinprog**		**lp_solve**	
**Dataset (# proteins)**	**MDS**	**CC-MDS**	**MDS**	**CC-MDS**
Combined (368)	6.5E-02 (76)	9.2E-09 (106)	2.1E-04 (90)	9.3E-09 (106)
Binary (358)	2.2E-03 (86)	1.7E-09 (109)	7.9E-08 (104)	1.7E-09 (109)
Co-complex (196)	4.0E-01 (44)	4.4E-06 (66)	5.6E-04 (59)	4.4E-06 (66v)

### Enrichment analysis of Gene Ontology terms

To indicate the biological significance of the predicted driver proteins, we compute the enrichment of them in each of the Gene Ontology terms using GO Term Finder [35,59]. All the three GO categories (BP, CC and MF) are considered. The Bonferroni correction is used to counteract the problem of multiple comparisons. A GO term is assumed to be statistically significantly enriched in a resulting set of driver proteins if the corresponding corrected p-value is lower than 0.01. In this section, we just consider the combined network and the intlinprog method for the CC-MDS model. This is because that the combined network has the highest recall and that the resulting CC-MDS proteins of the intlinprog and lp_solve methods are comparable.

We observe that the number of GO terms significantly enriched in CC-MDS proteins is larger than the number corresponding to MDS proteins (Additional file 2). This indicates that CC-MDS proteins are more functionally consistent than MDS proteins. Interestingly, all GO terms enriched in MDS proteins are also enriched in CC-MDS proteins. Therefore, functionally important proteins which can be captured by the standard MDS model can also be captured by the CC-MDS model. Biological functions with significant enrichments in CC-MDS proteins include many processes critical for normal cellular functioning, such as cell surface receptor signaling pathway, response to stimulus, single organism signaling, regulation of biological process, regulation of cellular process, cell death, defense response, gene expression, apoptotic process, T cell costimulation, leukocyte cell-cell adhesion etc. For detailed information, please refer to Additional file 2. Looking further into these results, we rank CC-MDS proteins according to the number of annotated GO terms in descending order. Here we only consider the terms that are significantly enriched in the resulting set of CC-MDS proteins. The complete ranked list is available as Additional file 3. We find that the CC-MDS proteins involved with more GO terms are more likely to be essential genes, aging genes, disease-associated genes, virus-targeted genes, transcription factors and protein kinases (Spearman’s rank correlation coefficient, p-value < 0.01). For example, out of the top 1% (e.g., 15) CC-MDS proteins, 12 of them are essential genes, 9 of them are aging genes, 6 of them are virus-targeted genes, and 6 of them are protein kinases (Table 9).

**Table 9:** **The top 1% (e.g., 15) CC-MDS proteins in the combined network**

**Protein**	**# GO terms**	**E**	**A**	**GD**	**OD**	**UD**	**VT**	**TF**	**PK**
AKT1	326	x	x	x	x	x			x
EGFR	315	x	x	x	x	x			x
MAPK3	308		x						x
TGFB1	302	x	x	x	x	x			
CDK1	293	x	x				x		
SIRT1	292	x	x						
CTNNB1	289	x	x	x	x	x			
MAPK1	284	x					x		x
RPS27A	279						x		
INS	272	x	x	x	x	x			
UBC	272	x							
IGF1	269	x	x	x	x	x			
LYN	269					x	x		x
TRAF6	267	x					x		
SRC	264	x			x	x	x		x

If a protein is an essential (“E”) gene, aging (“A”) gene, GAD disease (“GD”) gene, OMIM disease (“OD”) gene, Uniport disease (“UD”) gene, virus-targeted (“VT”) gene, transcription factors (“TF”) or protein kinase (“PK”), there is an “x” in the corresponding entry.

### Comparison with previous models

Before our study, Milenković et al. also developed a heuristic algorithm to identify dominating sets in PPI networks using the degree centrality and graphlet degree centrality [24]. According to the centralities they used, their methods are referred to as “dominating sets-degree centrality” (DS-DC) and “dominating sets-graphlet degree centrality” (DS-GDC). They found that the predicted dominating sets could capture biological central genes. Therefore, it is interesting to compare the CC-MDS model with the DS-DC and DS-GDC models.

**Figure 8:**
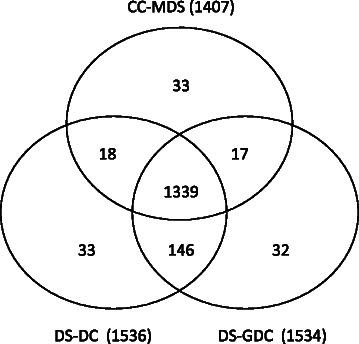
Overlap of the three sets of driver proteins produced by CC-MDS, DS-DC and DS-GDC algorithms applied on the combined network.

We run the three models on the three considered networks. We find that the CC-MDS model produces smaller dominating sets than the DS-DC and DS-GDC models and the overlaps between the three resulting dominating sets are large (Figure 8, Figure S11 in Additional file 1). Therefore, compared to the dominating sets identified by the DS-DC and DS-GDC models, the ones identified by the CC-MDS model are minimal. These results also indicate that CC-MDS proteins can capture a huge portion of the dominating sets produced by the DS-DC and DS-GDC models. We do not present the results about the functional significance of dominating sets produced by the DS-DC and DS-GDC models in the manuscript for the following reasons. First, our main focus is to identify minimum dominating sets. However, the dominating sets identified by the DS-DC and DS-GDC models are larger than the ones identified by the CC-MDS model. Second, as we discussed before, the dominating sets produced by the three models overlap with each other considerably, therefore, their functional significance may be similar.

### Analysis of computational time

As mentioned above, both the MDS model and the CC-MDS model involve solving a binary integer-programming problem and the difference between them lies primarily in the objective function. Therefore, they have similar computational complexities in theory. Table 10 presents the time cost of the MDS and CC-MDS models solved using both the “intlinprog” and “lp_solve” algorithms. We implement the algorithms using Matlab in a workstation with Intel 4 CPU (3.40 GH × 4) and 16 GB RAM. We find that all methods can produce the dominating sets within 2 seconds. The time costs of the MDS model and the CC-MDS model are comparable when computed using the “intlinprog” method; while the CC-MDS model is more efficient than the MDS model when computed using the “lp_solve” method. Please note that here we only consider time cost of solving the optimization problems (Equations (1) and (2)). In practice, we also need some time to compute degree centrality and betweenness centrality when we use the CC-MDS model to determine driver proteins. Encouragingly, a lot of soft packages (e.g., MatlabBGL [32]) which can compute centralities of a given network efficiently have been developed. Therefore, compared to the MDS model, the CC-MDS model can capture more functional significant proteins without loss of efficiency.

**Table 10:** **Time cost (second) of the MDS and CC-MDS models**

	**intlinprog**		**lp_solve**	
**Dataset**	**MDS**	**CC-MDS**	**MDS**	**CC-MDS**
Combined	0.82	0.77	1.81	1.10
Binary	0.77	0.76	1.50	0.99
Co-complex	0.16	0.16	0.21	0.16

## Conclusions

In this paper, we study how to incorporate heterogeneous centralities (degree and betweenness) of proteins into the standard minimum dominating set model, providing a more effective way to determine driver proteins that play an important role in controlling the entire network. Even though the correction seems minor and innocuous, we experimentally find that the corrected version is less sensitive to the optimization methods than the uncorrected counterpart. Furthermore, the centrality-corrected model can detect significantly more proteins that carry important topological and functional characteristics than the original model.

The corrected-model presented here raise several questions, answers to which could further improve the performance. For example, although our centrality-corrected model can considerably increase the overlap between the sets of driver proteins computed using different optimization methods, there are still several algorithmic-dependent proteins in the combined and binary networks (see Table 2). We manually find that the proteins that can be only determined by one of method always have same interacting neighbors. Therefore theses proteins can not be distinguished using topological property alone. One possible solution is to use functional property (e.g., GO functions) of proteins to define the weights in Equation (3). By doing so, proteins would be more discriminative and proteins that carry out important biological functions could be predicted as driver proteins.

## Additional files

Additional file 1
**Supplementary tables and figures.** This section provides the supplementary tables and figures referred in the main text.

Additional file 2
**Enrichment analysis of Gene Ontology terms.** This section provides a table that presents the results of GO enrichment analysis of the predicted driver proteins in the combined network.

Additional file 3
**Complete ranked lists of CC-MDS proteins.** This section provides a table that presents a complete list of CC-MDS proteins ranked according to the number of associated GO annotations in the combined network.
