# Supplementary material for: Determining minimum set of driver nodes in protein-protein interaction networks
Source: BMC Bioinformatics. 2015 May 7;16:146. doi: 10.1186/s12859-015-0591-3 (PMC4428234; doi:10.1186/s12859-015-0591-3)
Supplement: Additional file 1 — Supplementary tables and figures. This section provides the supplementary tables and figures referred in the main text. [file 12859_2015_591_MOESM1_ESM.pdf]

Determining minimum set of driver nodes in protein-protein interaction networks: Additional file 1

Xiao-Fei Zhang, Le Ou-Yang, Yuan Zhu, Meng-Yun Wu, and Dao-Qing Dai

Contents

1 Supplementary Table **2**

2 Supplementary Figure **3**

1 Supplementary Table

Table S1: Significance of the difference between degree populations of predicted driver proteins and non-driver proteins

	intlinprog		lp_solve	
Dataset	MDS	CC-MDS	MDS	CC-MDS
combined	4.9E-10	0	1.6E-131	0
binary	2.3E-15	0	6.7E-183	0
co-complex	8.3E-05	1.3E-134	4.2E-64	1.3E-134

Table S2: Significance of the difference between betweenness populations of predicted driver proteins and non-driver proteins

	intlinprog		lp_solve	
Dataset	MDS	CC-MDS	MDS	CC-MDS
combined	1.6E-25	0	1.1E-235	0
binary	1.3E-30	0	4.6E-314	0
co-complex	1.1E-15	8.5E-248	9.4E-130	8.5E-248

Table S3: Significance of the difference between populations of the number of annotated protein complexes of predicted driver proteins and non-driver proteins

	intlinprog		lp_solve	
Dataset	MDS	CC-MDS	MDS	CC-MDS
combined	3.5E-03	2.3E-06	5.2E-05	2.2E-06
binary	2.3E-02	6.1E-06	5.1E-04	5.9E-06
co-complex	1.5E-02	5.6E-05	5.4E-04	5.6E-05

Table S4: Significance of the difference between populations of the number of annotated GO annotations of predicted driver proteins and non-driver proteins

		intlinprog		lp_solve	
Dataset	Ontology	MDS	CC-MDS	MDS	CC-MDS
combined	BP	2.9E-06	1.4E-33	6.3E-17	2.5E-33
	CC	7.8E-11	1.7E-44	6.7E-26	8.7E-44
	MF	1.5E-15	3.2E-78	8.1E-50	6.4E-78
binary	BP	3.1E-07	5.6E-31	2.9E-20	9.8E-31
	CC	1.3E-14	4.9E-43	1.8E-28	1.2E-42
	MF	1.8E-16	7.1E-72	3.8E-48	1.4E-71
co-complex	BP	1.9E-05	3.2E-28	7.8E-20	3.2E-28
	CC	4.4E-07	1.7E-31	2.0E-21	1.7E-31
	MF	5.0E-13	5.5E-75	1.1E-43	5.5E-72

2 Supplementary Figure

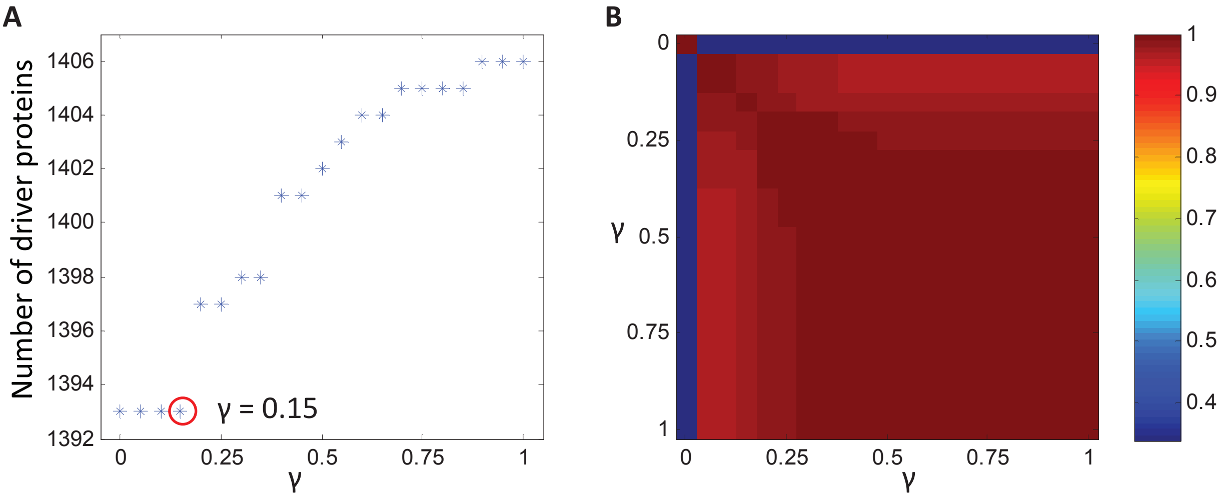

Figure S1: Effect of parameter γ on the resulting CC-MDS proteins for the intlinprog method in the binary network. In (A), we present the effect of parameter γ on the number of predicted driver proteins. The x-axis denotes the value of γ; the y-axis denotes the number of driver proteins determined using the CC-MDS model; the red circle labels the value of γ we choose. In (B), we present the overlap rate between the sets of driver proteins obtained using different values of γ.

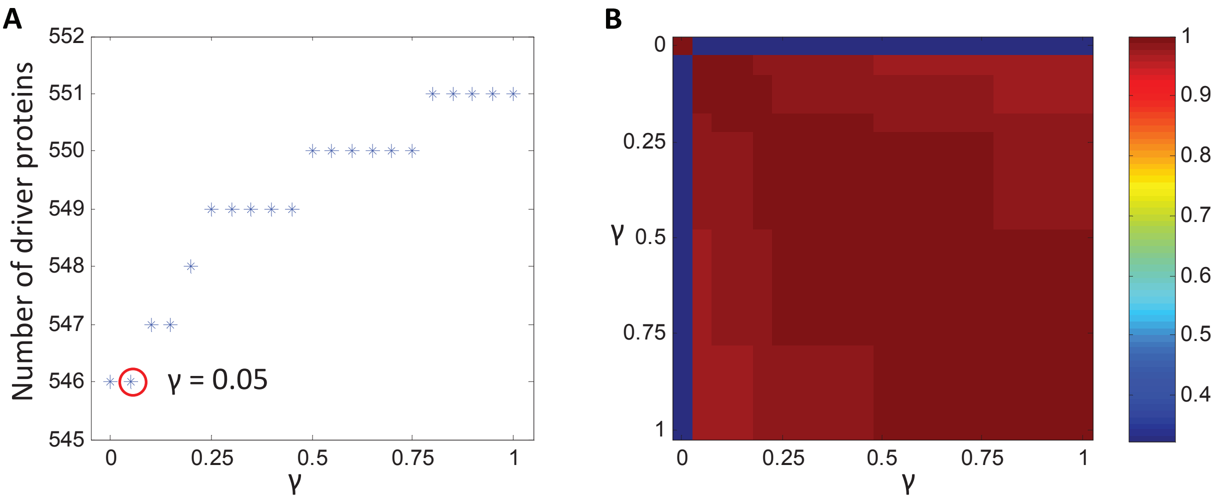

Figure S2: Effect of parameter γ on the resulting CC-MDS proteins for the intlinprog method in the complex network. In (A), we present the effect of parameter γ on the number of predicted driver proteins. The x-axis denotes the value of γ; the y-axis denotes the number of driver proteins determined using the CC-MDS model; the red circle labels the value of γ we choose. In (B), we present the overlap rate between the sets of driver proteins obtained using different values of γ.

A

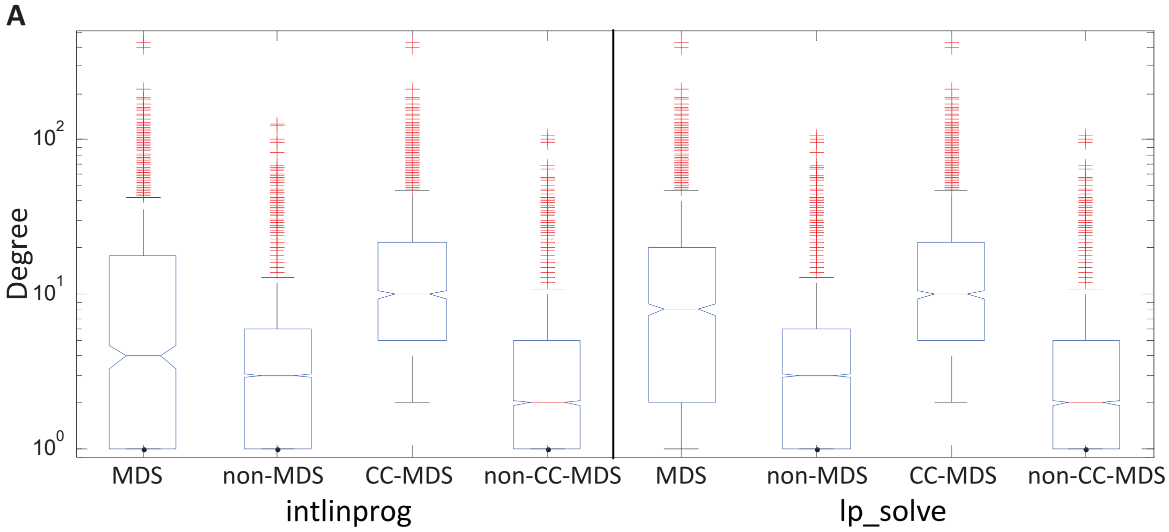

B

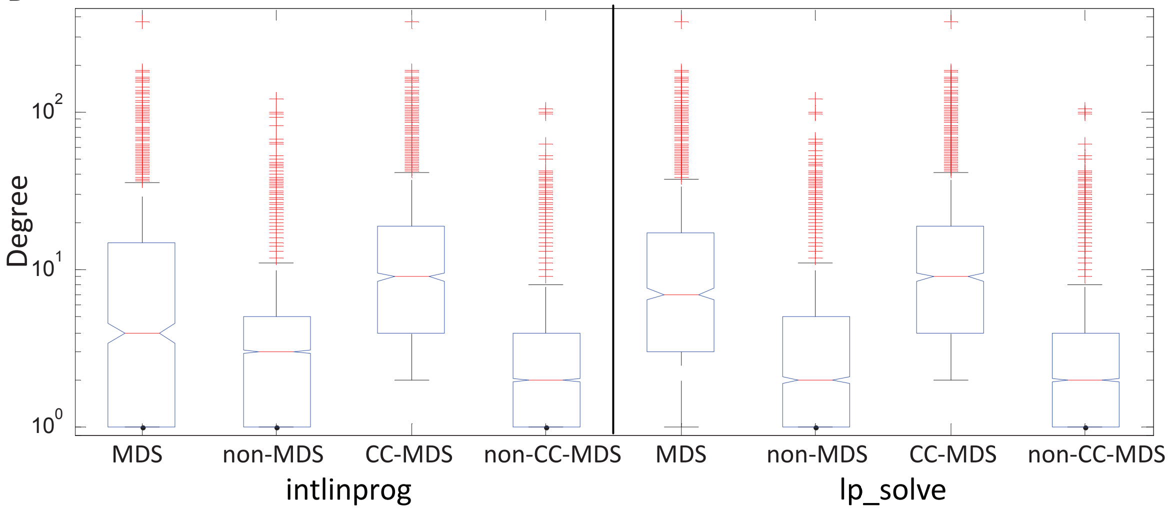

C

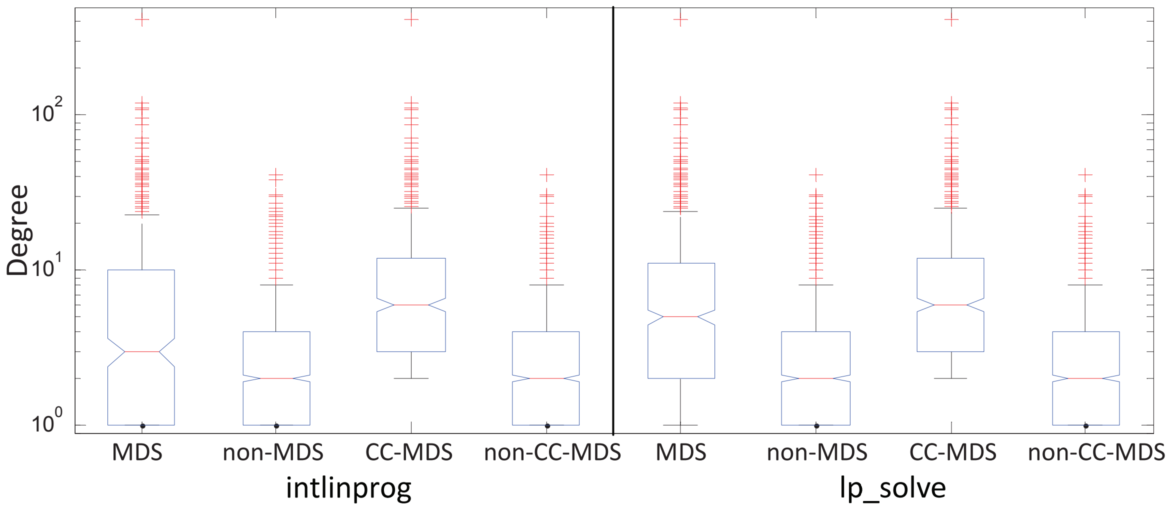

Figure S3: Degree distributions of predicted driver and non-driver proteins. The degree distributions of predicted driver and non-driver proteins are represented by box plots (line = median). (A) combined network; (B) binary network; (C) co-complex network.

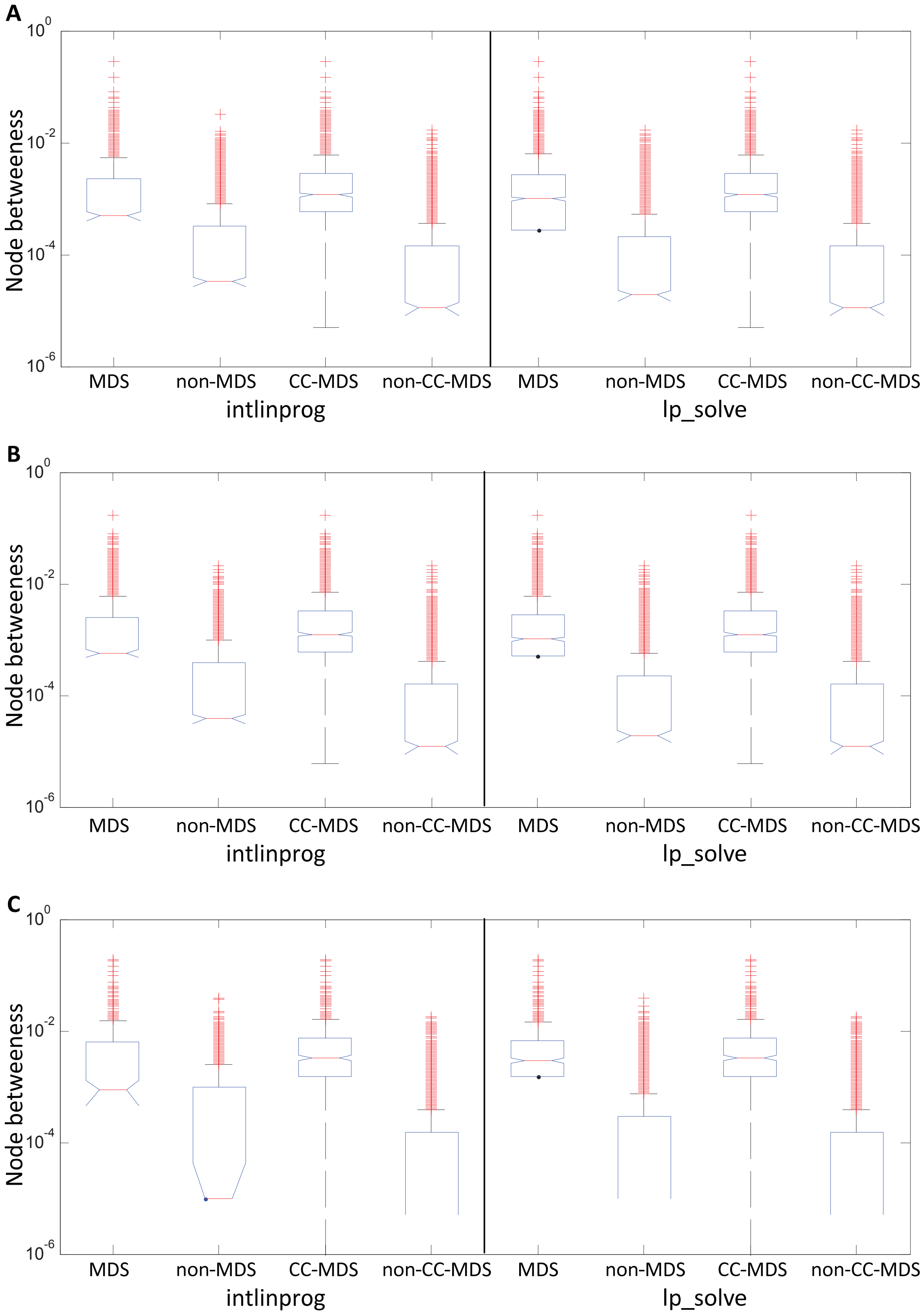

Figure S4: Betweenness distributions of predicted driver and non-driver proteins. The betweenness distributions of predicted driver and non-driver proteins are represented by box plots (line = median). (A) combined network; (B) binary network; (C) co-complex network.

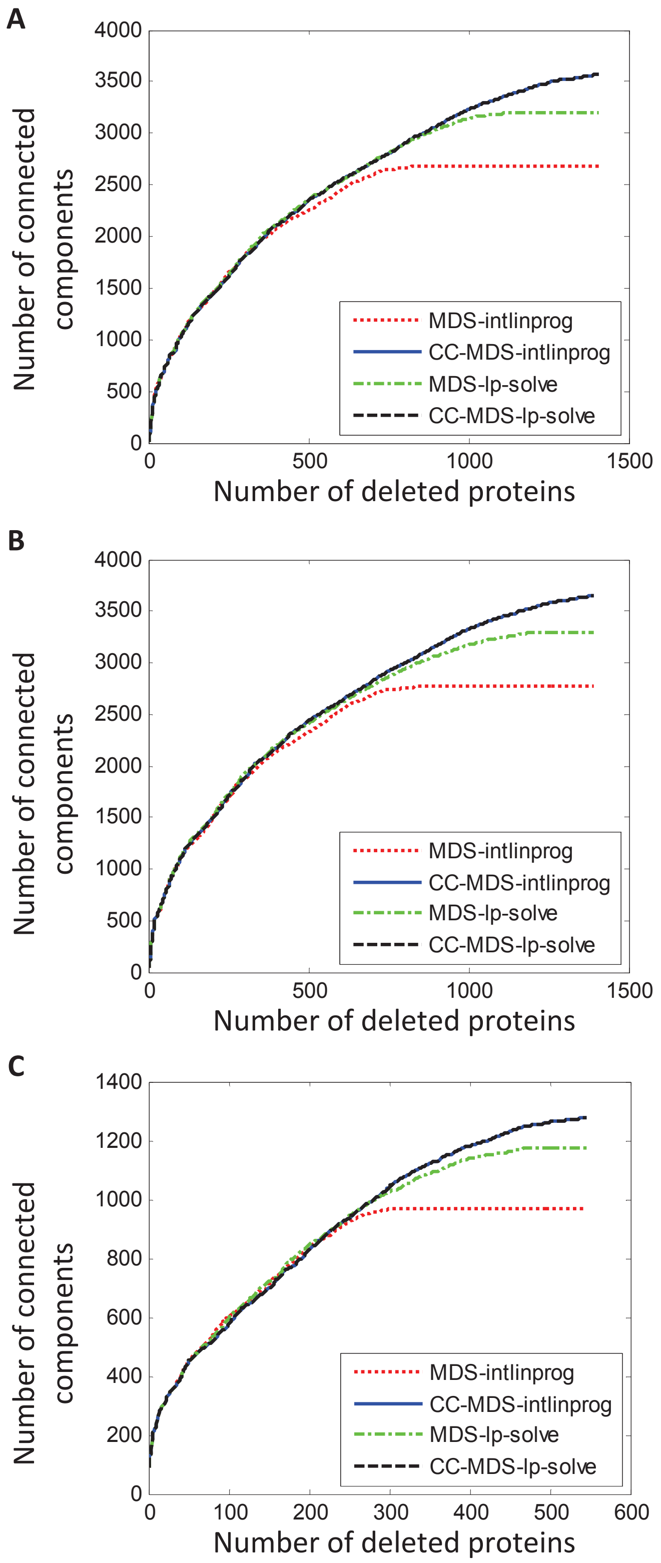

Figure S5: Vulnerability to attack against predicted driver proteins quantified using the number of connected components. Starting with the most connected proteins, the proteins are successively deleted and the number of connected components after each deletion is calculated. There is one curve for each set of predicted driver proteins that shows the number of connected components as a function of the number of deleted proteins. (A) combined network; (B) binary network; (C) co-complex network.

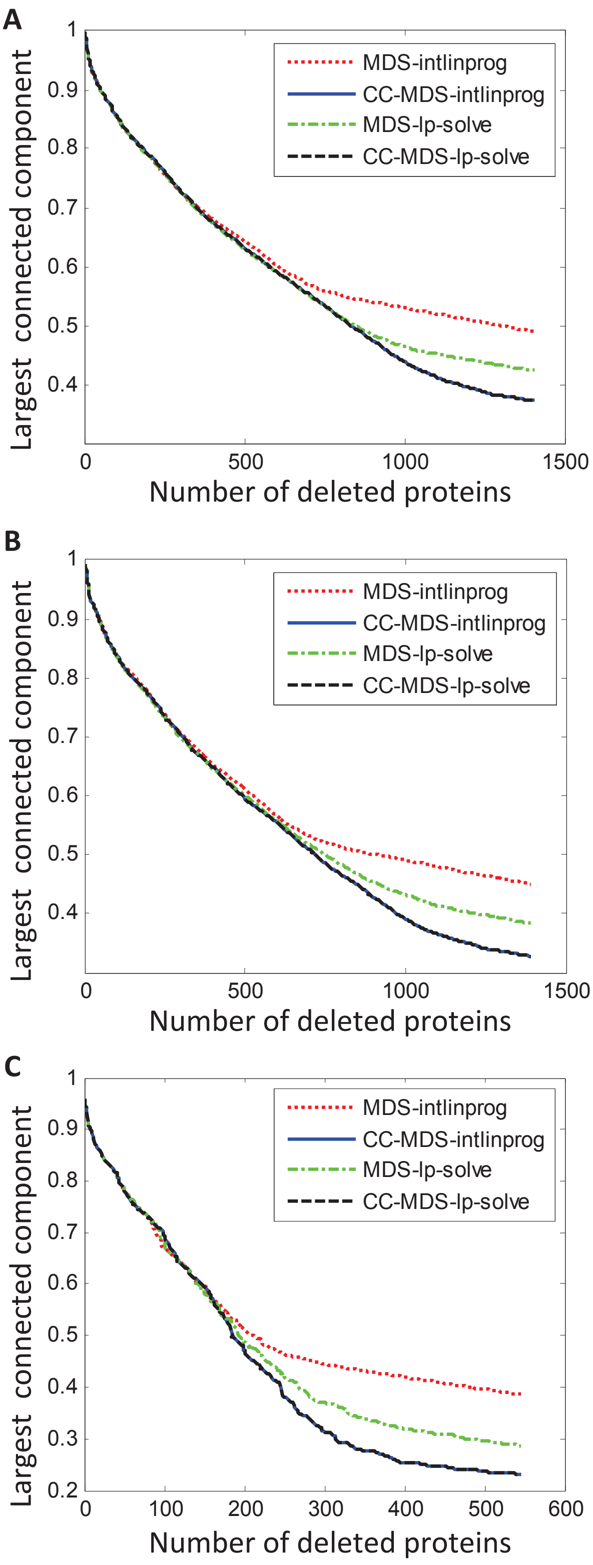

Figure S6: Vulnerability to attack against predicted driver proteins quantified using the largest connected component. Starting with the most connected proteins, the proteins are successively deleted and the size of largest connected component after each deletion is calculated. There is one curve for each set of predicted driver proteins that shows the fraction of nodes in the largest connected component as a function of the number of deleted proteins. (A) combined network; (B) binary network; (C) co-complex network.

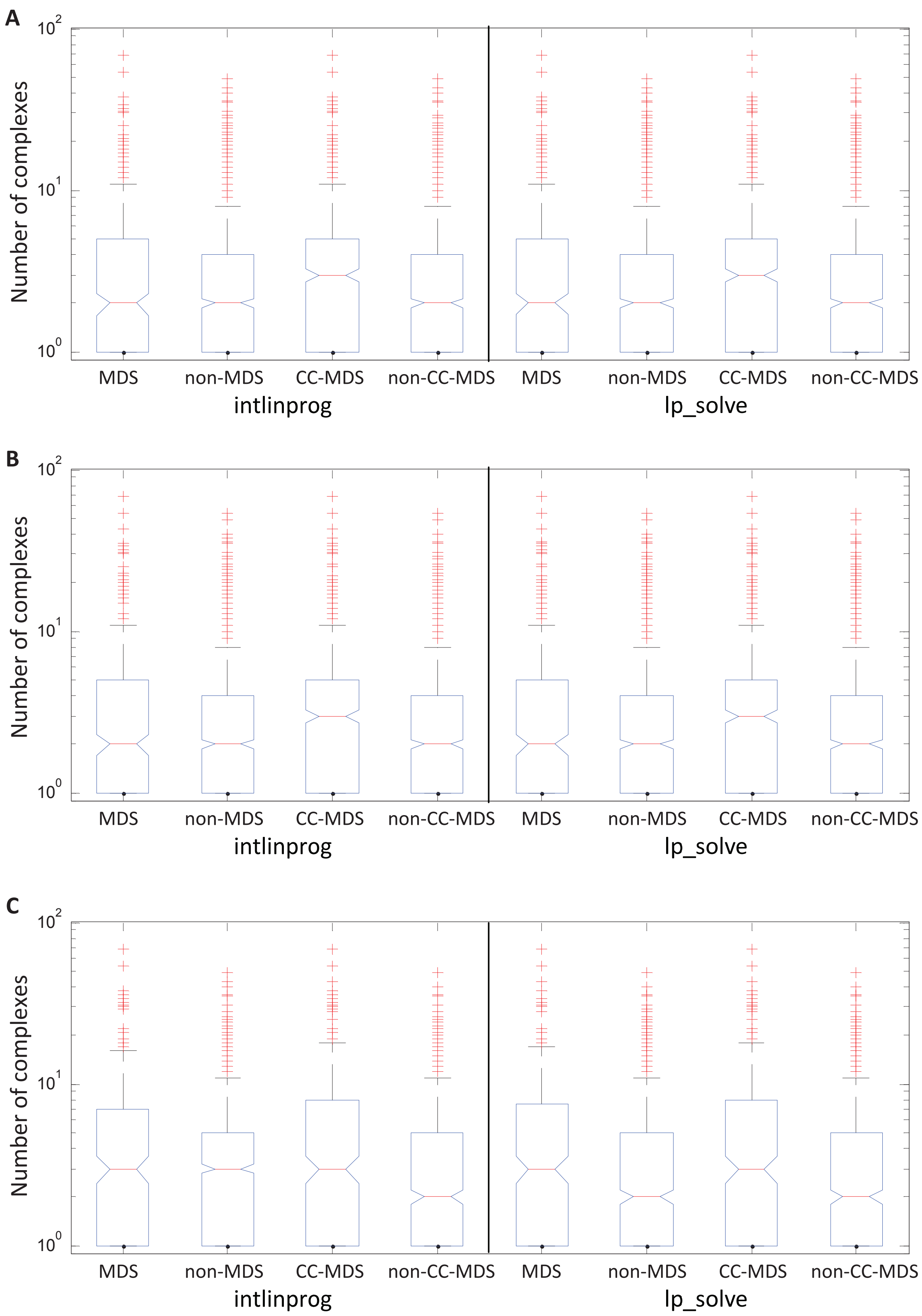

Figure S7: Distributions of the number of associated complexes of predicted driver and non-driver proteins. The distributions of the number of associated protein complexes of predicted driver and non-driver proteins are represented by box plots (line = median). (A) combined network; (B) binary network; (C) co-complex network.

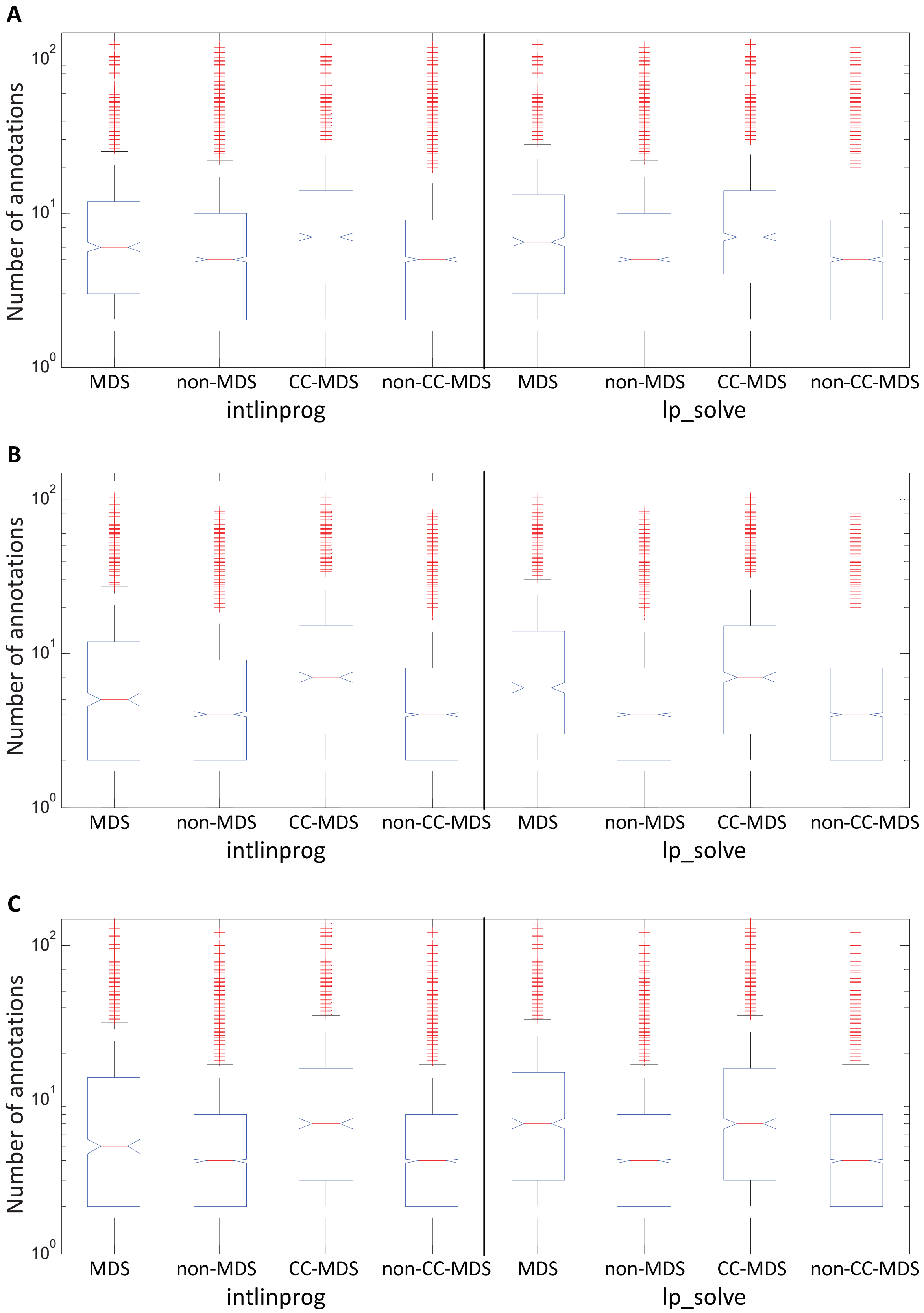

Figure S8: Distributions of the number of associated GO annotations of predicted driver and non-driver proteins in the combined network. The distributions of the number of associated GO annotations of predicted driver and non-driver proteins are represented by box plots (line = median). (A) biological process; (B) cellular component; (C) molecular function.

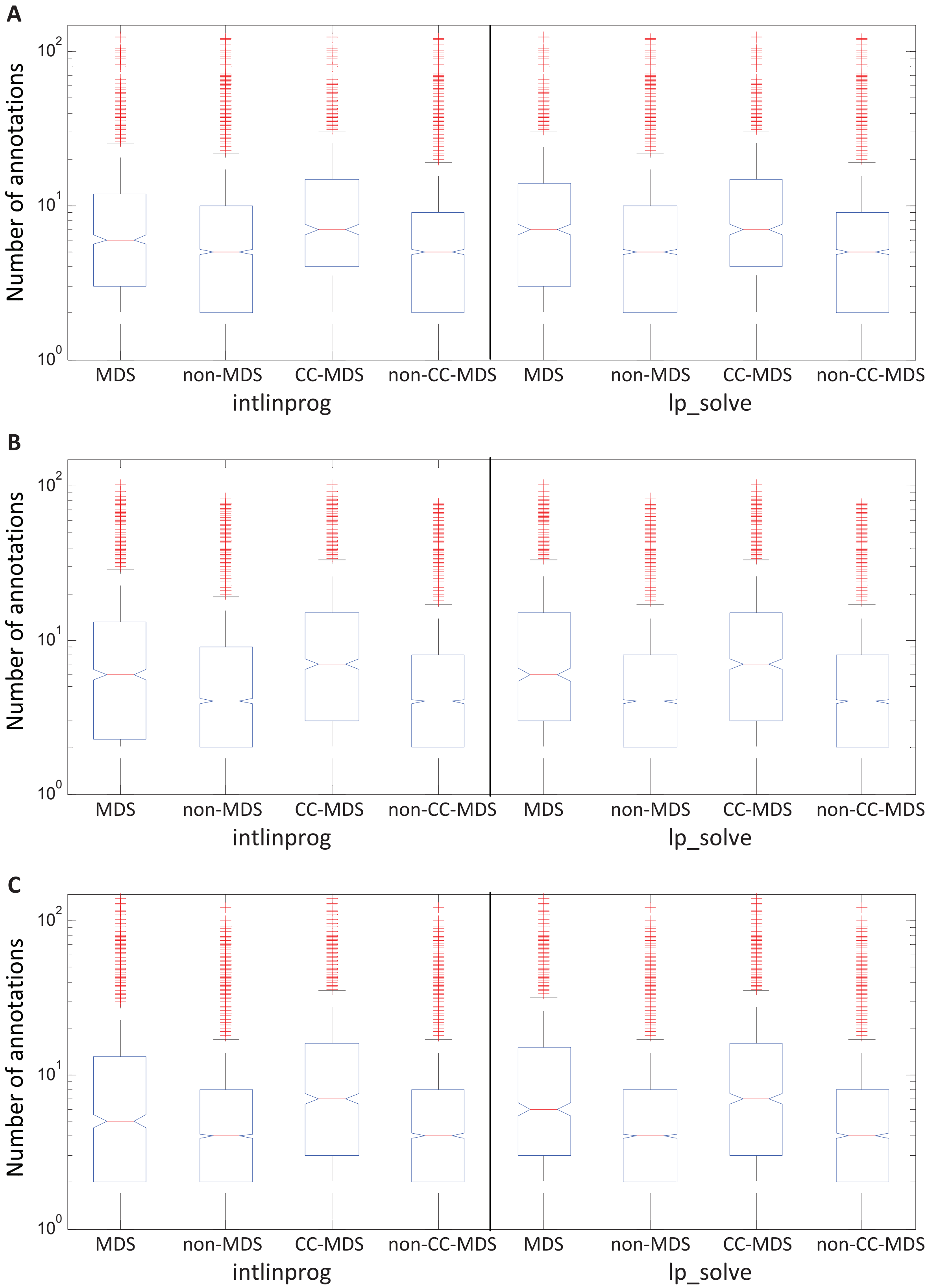

Figure S9: Distributions of the number of associated GO annotations of predicted driver and non-driver proteins in the binary network. The distributions of the number of associated GO annotations of predicted driver and non-driver proteins are represented by box plots (line = median). (A) biological process; (B) cellular component; (C) molecular function.

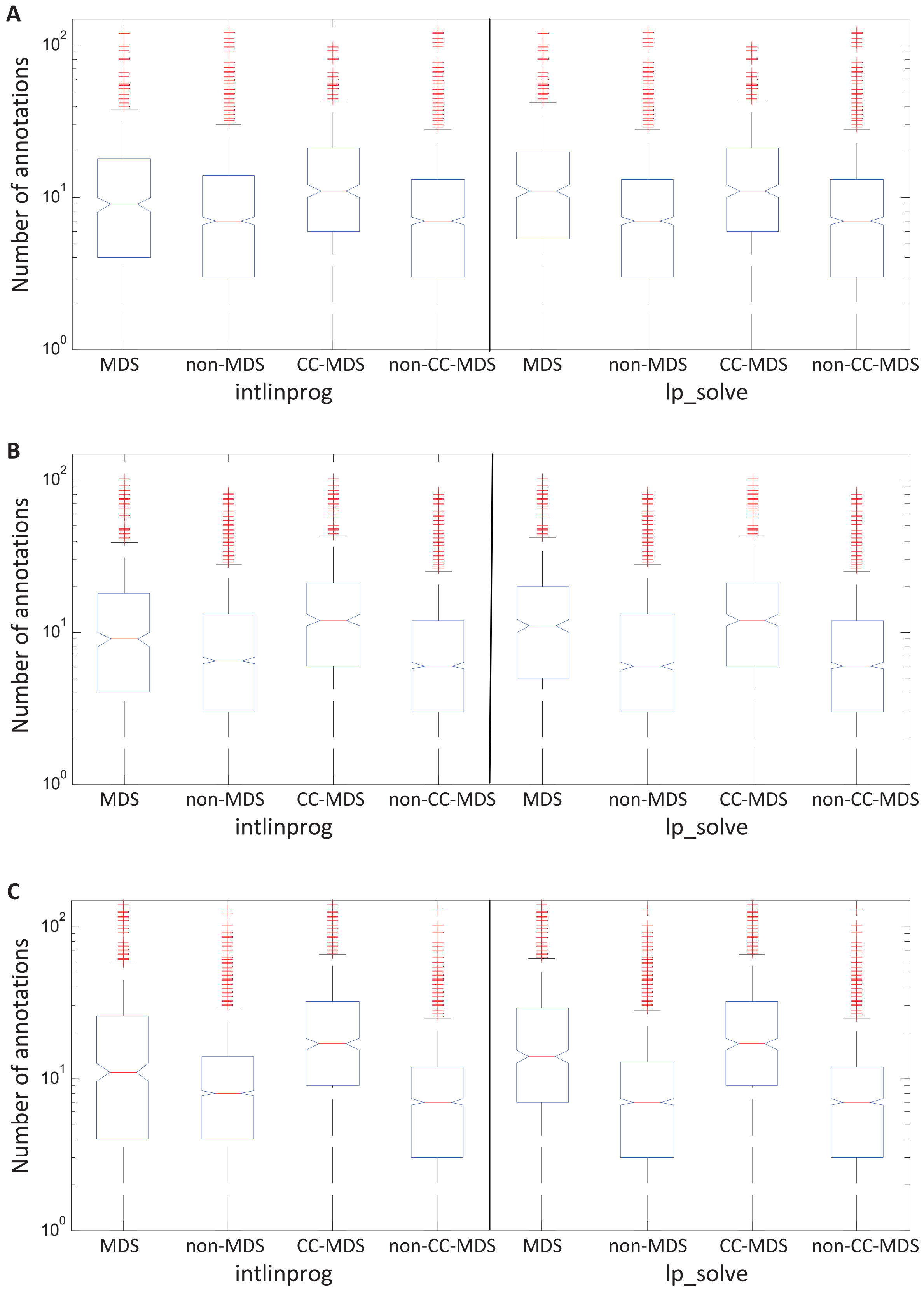

Figure S10: Distributions of the number of associated GO annotations of predicted driver and non-driver proteins in the complex network. The distributions of the number of associated GO annotations of predicted driver and non-driver proteins are represented by box plots (line = median). (A) biological process; (B) cellular component; (C) molecular function.

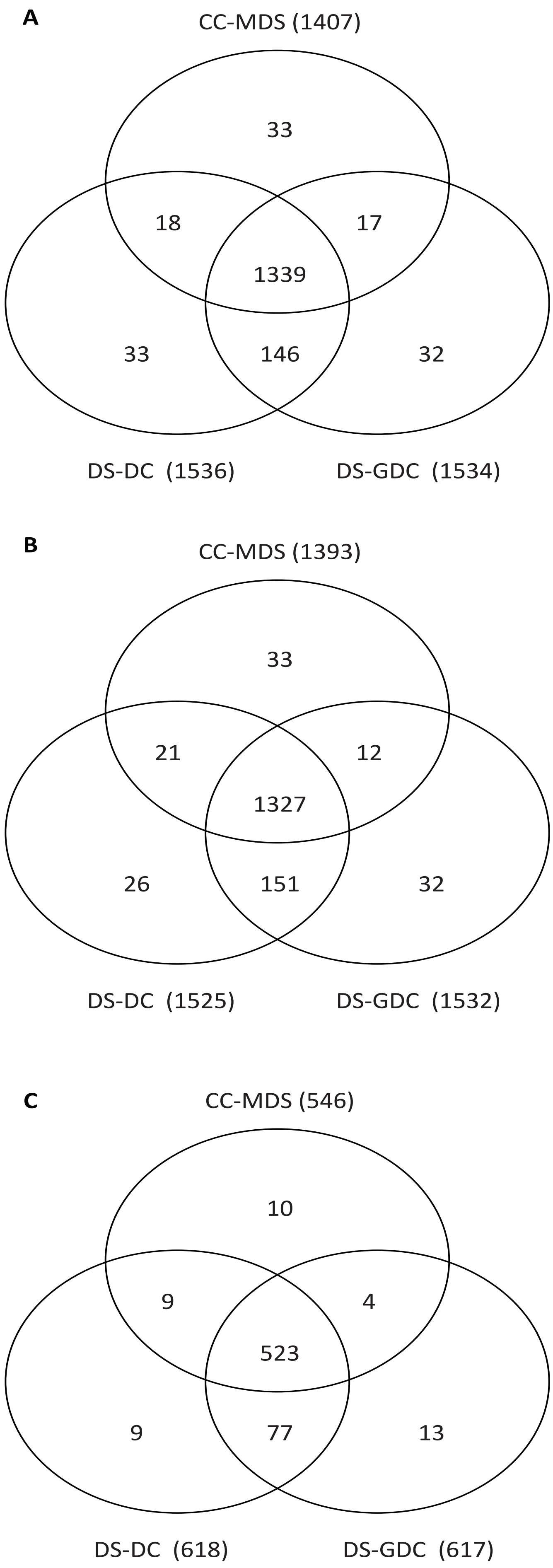

Figure S11: Overlap of the three sets of driver proteins produced by CC-MDS, DS-DC and DS-GDC algorithms applied on the three networks considered. (A) combined network; (B) binary network; (C) co-complex network.
